# Differential expression of apoptosis-related factors induces the age-related apoptosis of the gracilis muscle in humans

**DOI:** 10.3892/ijmm.2014.1675

**Published:** 2014-02-27

**Authors:** SOO YEON PARK, JUNG HWAN LEE, HA YOUNG KIM, KYOUNG HO YOON, SEONG KYU PARK, MUN SEOG CHANG

**Affiliations:** 1 Graduate School of Education, Yong In University, Seoul 130-701, Republic of Korea; 2 Department of Prescriptionology, College of Korean Medicine, Seoul 130-701, Republic of Korea; 3 Department of Orthopaedic Surgery, School of Medicine, Kyung Hee University, Seoul 130-701, Republic of Korea

**Keywords:** human skeletal muscle, gracilis, apoptosis, aging, apoptosis-inducing factor

## Abstract

In the normal aging process, apoptosis has been implicated as a mechanism responsible for the loss of muscle cells and plays an important role in age-related muscle loss. Several signaling pathways involved in skeletal muscle apoptosis are currently under intense investigation, particularly the caspase-independent pathway. This study investigated the age-related apoptotic changes occurring in the gracilis muscle in humans between 10 and 50 years of age. For this purpose, muscle samples were divided into 5 groups (n=8). Terminal deoxynucleotidyl transferase-mediated dUTP nick end-labeling (TUNEL) staining and immunofluorescence detection were performed to determine the number of apoptotic muscle cells in each group. In addition, the expression levels of apoptosis-related factors, such as Bcl-2, Bax, apoptosis-inducing factor (AIF), caspase-3 and calpain-1 were determined by RT-PCR and western blot analysis. TUNEL assay revealed a significant increase in gracilis muscle apoptosis with aging. The activity of caspase-3 in the gracilis muscle tended to change with age, although the changes were not significant, while the increase in DNA nuclei in muscle from 50 years of age (5.419±0.97) was associated with an increase in the expression of AIF, as observed both at protein (10–30%) and mRNA level (10–60%) in gracilis tissues. Taken together, our results demonstrated that the relative Bcl-2 expression decreased with aging, while Bax expression was upregulated compared to 10 -year-olds. In addition, a double-labeling experiment with TUNEL staining and immunofluorescence revealed the co-localization of nuclear AIF-positive and TUNEL-labeled cells. This study suggests that apoptosis in gracilis skeletal muscle in the elderly is partly mediated through the expression of Bcl-2/Bax and the degradation of AIF.

## Introduction

Human skeletal muscle is often injured physically or chemically and changes occur with aging and disease. The age-related loss of muscle mass, strength and quality, referred to as sarcopenia, is a common feature of aging that is characterized by a decline in both the number and size of muscle fibers (1–3). With these age-related changes, the estimated rate of muscle loss is 1–2% per year after the age of 50 years (4,5). The loss of muscle mass due to apoptosis with normal aging has been investigated in a number of studies (6,7).

Apoptosis, a process of individual cell death regulated by the activation of specific genes, is an important regulatory process that occurs during normal development and in the progression of specific diseases (8). Although apoptosis may occur via several mechanisms (9,10), one mechanism involves external factors that bind to membrane death receptors outside the cell, and another involves internal cellular events that lead to the release of specific cell death molecules from the mitochondria (11–13). In the mitochondrial-mediated pathway, in response to cellular stress or DNA damage, p53 can induce apoptosis by regulating the proteins of the Bcl-2 family and by translocating to the mitochondria and activating apoptotic signaling directly (14–16). In addition, caspase-3 can activate caspase-activated DNase, leading to DNA fragmentation and cell death (17). Caspase-independent mechanisms also exist, such as the release of apoptosis-inducing factor (AIF) and endonuclease G (EndoG) from the mitochondria, inducing large-scale DNA fragmentation (18–21).

The release of apoptosis-inducing factors by the mitochondria, nuclear translocation and DNA agmentation associated with AIF have been demonstrated in several systems and cell types. AIF is translocated to the nucleus after being released from the mitochondria, inducing DNA fragmentation (22–24). AIF is found in several human tissues, including cardiac and skeletal muscle (25,26). In addition, the calcium-dependent proteinase (calpain) system is present in every vertebrate cell. At least 3 calpains exist in humans: calpain-1 (μ-calpain), calpain-2 (m-calpain) and calpain-3 (n-calpain, p94) (27,28). A number of studies have demonstrated that the AIF and EndoG pro-apoptotic factors, which are released from the mitochondria by calpain activity, are upregulated in sarcopenic muscle (29–32). However, whether this causes the apoptosis that occurs with the normal aging process in human muscle is not known. Previously, we reported the age-dependent induction of AIF in the human semitendinosus skeletal muscle (33). The aim of the present study was to investigate the general pattern of skeletal muscle apoptosis, particularly in the human gracilis skeletal muscle with extended age (up to 50 years old). We examined the expression of apoptosis-related factors to elucidate the key players associated with the aging-related process in muscles from 10- and 50-year-old individuals.

## Materials and methods

### Muscle sampling

Samples of gracilis skeletal muscle were collected from individuals of different ages (10, 20, 30, 40 and 50 years old) who underwent anterior cruciate ligament reconstruction with gracilis. Muscles were harvested from individuals of 10 to 50 years of age and 8 samples were analyzed in each age group. All the human subjects were healthy with no muscle-related clinical conditions. Muscle tissues were prepared from the musculotendinous junction, mounted, immediately frozen in liquid nitrogen and stored at −80ºC for immunohistochemical and biochemical analyses. Ethical consent was obtained from the Kyung Hee Medical Center Institutional Review Board.

### Histological analysis

Different human gracilis skeletal muscle tissues (n=8 per age group) were fixed in 4% paraformaldehyde, embedded in optimum cutting temperature (OCT) compound, divided into 15-μm-thick sections and stained with hematoxylin and eosin (H&E).

### In situ terminal deoxynucleotidyl transferase-mediated dUTP nick end-labeling (TUNEL)

TUNEL assays were performed to detect DNA strand breaks using a commercial kit following the instructions provided by the manufacturer (Chemicon International, Temecula, CA, USA). Briefly, 15-μm-thick sections of skeletal muscle (n=8 per age group) were mounted onto Silane-coated glass slides. The dehydrated sections were treated with 20 μg/ml DNase-free proteinase K (Sigma-Aldrich Corp., St. Louis, MO, USA) to retrieve antigenic epitopes, followed by 3% H_2_O_2_ to quench endogenous peroxidase activity. Free 3′-OH termini were labeled with digoxigenin-dUTP for 1 h at 37ºC utilizing a terminal deoxynucleotidyl transferase reaction mixture. The incorporated digoxigenin-conjugated nucleotides were detected using a horseradish peroxidase-conjugated anti-digoxigenin antibody and 3,3′-diaminobenzidine. The dehydrated sections were cleared in xylene, mounted with Canada balsam and enclosed with coverslips.

### Immunohistochemistry

Sections (15-μm-thick) of frozen muscle tissue (n=8 per age group) were mounted onto Silane-coated glass slides and fixed in 4% paraformaldehyde (Sigma-Aldrich Corp.) for 1 h at 4ºC and endogenous peroxidase activity was blocked by the immersion of the sections in 3% H_2_O_2_ in 100% methanol for 15 min. All samples were incubated in 10% normal donkey serum (NDS) in phosphate-buffered saline (PBS) for 1 h at room temperature and were incubated with antibodies AIF (diluted 1:100; Cell Signaling Technology, Inc., Danvers, MA, USA). Immunohistochemical procedures used antibodies from several sources to establish antibody specificity and confirm immunostaining and protein expression. Primary antibody binding was visualized using Cy3-labeled donkey anti-rabbit antibody (1:500; Jackson ImmunoResearch Inc., West Grove, PA, USA). After staining, the sections were mounted with mounting medium with DAPI.

### Extraction of total RNA and reverse transcriptase PCR

Frozen gracilis skeletal muscle was homogenized on ice in 1 ml of ice-cold TRIzol reagent (Invitrogen Corp., Carlsbad, CA, USA). First-strand cDNA synthesis with 5 μg of total RNA was performed using MMLV reverse transcriptase and oligo(dT) primers for 1 h at 42ºC. Subsequently, the PCR amplification was performed by a modified method originally described in the study by Saiki *et al* (34). Total RNA was solubilized in RNase-free H_2_O and quantified twice by measuring the optical density (OD) at 260 nm. cDNA was synthesized from 2 g of total RNA, and reverse transcription (Promega Corp., Madison, WI, USA) was performed at 42ºC for 1 h following incubation at 95ºC for 5 min. cDNA amplification was carried out according to the following procedure: 95ºC for 1 min, 56ºC (β-actin), 58ºC (AIF, caspase-3, Bacl-2, Bax and calpain-1) for 1 min, 72ºC for 1 min. Twenty-six to 40 cycles were run, and the reaction was prolonged for 10 min at 72ºC. The sequences of the primers used for PCR were as follows: AIF forward, 5′-AGACGATCCCAAA TAATGCAG-3′ and reverse, 5′-TAGCTCTAGGTGAG TCTTGG-3′; caspase-3 forward, 5′-CGAAATTCAAA GGATGGCTCCTGGTT-3′ and reverse, 5′-CGGTTAA CCCGGGTAAGAAATGTGCAT-3′; Bcl-2 forward, 5′-GCA CGCTGGGAGAAAGGGTACGAT-3′ and reverse, 5′-CACA TCTCCAGCATCCCACTCGTA-3′; Bax forward, 5′-TGCC TCAGGATGCGTCCACCAA-3′ and reverse, 5′-CGGC AATCATCCTCTGCATGCTCCAT-3′; calpain-1 forward, 5′-CATGGTGCTGACCAAGATGAAGGAGAT-3′ and reverse, 5′-GCGCAGCCGCCTCACGGCTCCCAGCCT GTT-3′; and β-actin forward, 5′-TCATGAGTGTGACG TTGACATCCGT-3′ and reverse, 5′-CCTAGAAGCATTT GCGGTGCACGATG-3′. The PCR products were separated on 1.5% agarose gels, visualized by ethidium bromide staining using the i-MAX gel image analysis system (CoreBioSystem, Seoul, Korea), and analyzed using Alpha Ease™ FC software (Alpha Innotech Corp., San Leandro, CA, USA).

### Western blot analysis

Western blot analyses were performed to detect AIF and caspase-3 expression on muscle tissue (n=8). Muscle samples were placed in loading buffer, boiled for 5 min and centrifuged. Following quantification, the supernatants were loaded on a 10% sodium dodecylsulfate-polyacrylamide gel and subjected to electrophoresis. The fractionated proteins were transferred onto a polyvinylidene fluoride (PVDF) membranes (Milipore, Billerica, MA, USA), and the membranes, after blocking in 10% non-fat dry milk in TPBS buffer for 1 h at room temperature, were incubated with the primary antibodies to AIF (diluted 1:1,000), caspase-3 (diluted 1:1,000) and β-tubulin (diluted 1:1,000) (all from Cell Signaling Technology, Inc.) and then for 2 h with horseradish peroxidase-conjugated secondary antibodies (1:500; Jackson ImmunoResearch Inc.). After intervening washes, the membranes were developed with the ECL western blotting detection system (Thermo Fisher Scientific, Inc., Waltham, MA, USA) and the resulting chemilumnescence was exposed to film (Agfa HealthCare, Greenville, SC, USA). A tonsil served as a positive control.

### Statistical analysis

Statistical analysis was performed using GraphPrism 4.0.3 software (GraphPad Software, Inc., San Diego, CA, USA). All data are presented as the means ± standard deviation (SD) and a Student’s t-test was used to compare group means. AIF antibody and TUNEL assay were observed under a light microscope at magnification (×400). Images were captured using a Zeiss fluorescent microscope and myofibers were counted and measured using Axiovision 4 software (Carl Zeiss MicroImaging GmbH, Jena, Germany).

## Results

### Muscle sampling and histological analysis

**Figure 1:**
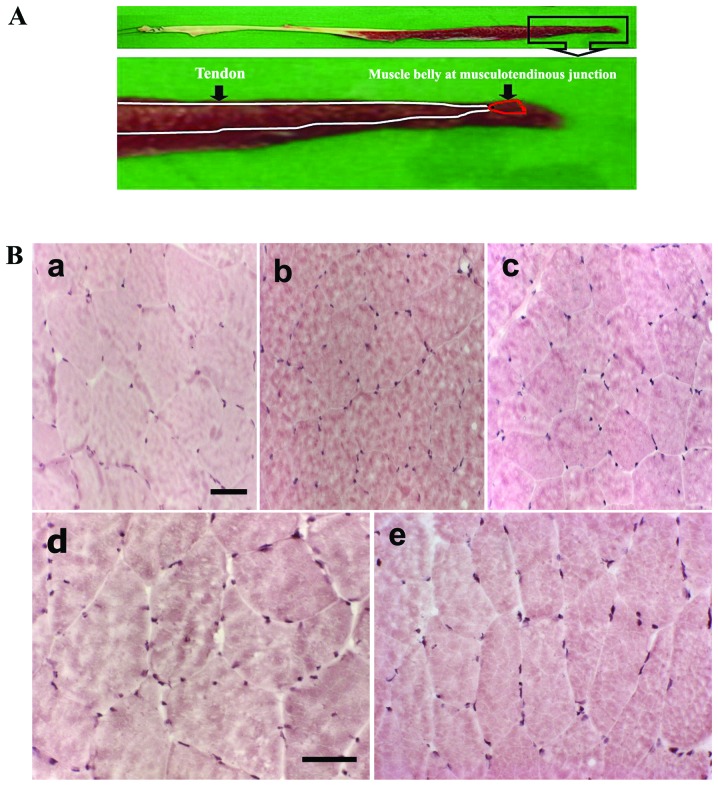
Gracilis muscle sampling and staining of morphological changes in gracilis skeletal muscle. (A) Muscle tissues sampling were prepared from the musculotendinous junction. (B) Staining of morphological changes in frozen sections of gracilis skeletal muscle using the hematoxylin and eosin (H&E). (a) Individuals of 10 years of age, (b) 20 years of age, (c) 30 years of age, (d) 40 years of age, and (e) 50 years of age. Images were obtained at an objective magnification of ×200. Scale bars, 150 μm.

Samples of gracilis skeletal muscle were collected from the musculotendinous junction of different individuals (Fig. 1A). We used H&E staining to examine the morphological changes induced by apoptosis from aging in human gracilis skeletal muscle (Fig. 1B). The detection of large numbers of nuclei is a distinct feature of necrosis. With H&E staining, none of the tissues showed evidence of necrosis.

### Detection of apoptosis

**Figure 2:**
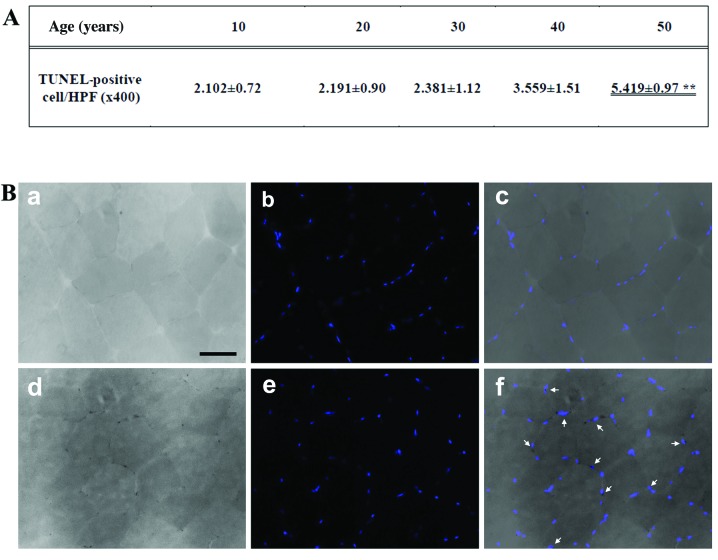
(A) DNA fragmentation in human gracilis skeletal muscle. Staining of fragmented DNA in frozen sections of gracilis skeletal muscle using the terminal deoxynucleotidyl transferase-mediated dUTP nick end labeling (TUNEL) assay. (a-c) Individuals of 10 years of age, and (d-f) 50 years of age. (a and d) TUNEL-positive nuclei, (b and e) DAPI-positive nuclei, (c and f) overlay of TUNEL and DAPI. Arrows indicate TUNEL-positive nuclei. Images were obtained at an objective magnification of ×200. Scale bars, 150 μm. (B) The number of TUNEL-positive nuclei to the gross area of nuclei as estimated by TUNEL staining was expressed as the TUNEL index. Data are presented as the means ± SE. ^**^P<0.001, compared with muscles from individuals of 10 years of age.

We performed TUNEL staining of the gracilis muscle sections. The size (mm^2^) of each area containing TUNEL-positive cells and the distance from the border of the infarction core were measured with a microruler under ×200 magnification. The number of apoptotic cells was counted in 3 high-power fields (HPF; ×400) under a light microscope, and the mean was recorded as cells/HPF (Fig. 2A). The number of TUNEL-positive nuclei increased with age, and was significantly higher in the muscles of the individuals of 50 years of age than the muscles of those who were 10 years of age (Fig. 2B).

### Changes in mRNA expression of apoptosis-related factors

**Figure 3:**
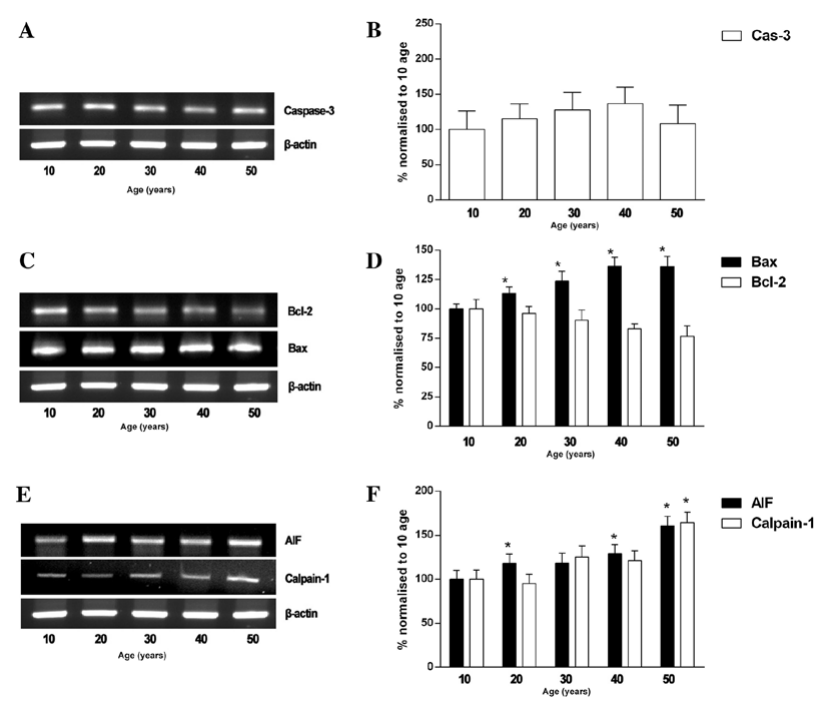
mRNA expression levels of AIF, calpain-1, Bcl-2, Bax and caspase-3 in human gracilis skeletal muscle of individuals of 10 to 50 years of age (n=8). (A–C) Representative immunoblots of AIF, calpain-1, Bcl-2, Bax and caspase-3. (D–F) Quantitative analysis of AIF, calpain-1, Bcl-2, Bax and caspase-3. Quantification of polymerase chain reaction (PCR) signals obtained using a densitometric analysis of the signal product optical density (OD). The bands were quantified by normalization to those from the individuals of 10 years of age. ^*^P<0.05, normalized to muscles of individuals of 10 years of age. Cas-3, caspase-3.

Since caspase-3 and AIF play pivotal roles in apoptosis, we investigated whether their activity is increased in gracilis muscle with age. The caspase-3 mRNA levels tended to increase with age; however, these changes were not significant (Fig. 3A and B). On the other hand, the gracilis muscle in individuals who were 10 and 50 years of age was found to have elevated levels of Bax (130±8%) and the relative mRNA expression level of Bcl-2 (76±8%) in the gracilis muscle declined significantly with age (Fig. 3C and D). We investigated whether a caspase-independent mechanism is involved in the increased apoptosis in the muscles of 50-year-olds. Our results revealed that the relative AIF mRNA level increased with age, and was 100±10, 118±11, 130±10, 139±11 and 160±11% in the individuals who were 10, 20, 30, 40 and 50 years of age, respectively. The expression of AIF significantly correlated with the mRNA expression of calpain-1 in muscle, which was 100±11, 95±10, 125±12, 121±11 and 164±11%, in the individuals who were 10, 20, 30, 40 and 50 years of age, respectively (Fig. 3E and F).

### Changes in expression of AIF in gracilis muscle of individuals of 50 years of age

**Figure 4:**
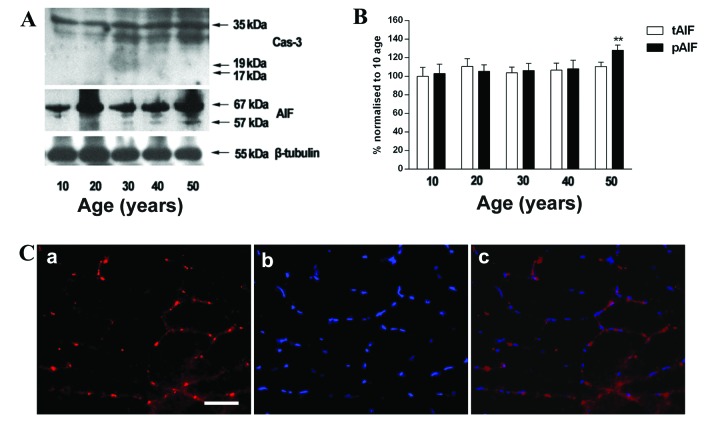
Expression of apoptosis-inducing factor (AIF) in the human gracilis skeletal muscle of individuals of 10 and 50 years of age (n=8). (A) Representative immunoblots of AIF. (B) Quantitative analysis of AIF. The western blot analysis bands were quantified by normalization to those from individuals of 10 years of age. (C) Expression AIF immunoreactivity is observed in gracilis skeletal muscle of individuals of 50 years of age (a) AIF-positive muscle fibers, (b) DAPI-positive muscle fibers, (c) overlay of AIF and DAPI. Images were obtained at an objective magnification of ×200. Scale bars, 150 μm. Cas-3, caspase-3; tAIF, total AIF; pAIF, phosphorylated AIF.

The relative expression of AIF was higher in the individuals who were 50 years of age than those who were 10 years of age (Fig. 4B), while the protein level of cleaved caspase-3 was not observed in the gracilis muscle (Fig. 4A). In addition, AIF was stained with a specific anti-AIF antibody and merged with DAPI staining to determine whether AIF co-localizes in the nuclei (Fig. 4C).

### Association between the number of AIF-positive cells and apoptotic nuclei

**Figure 5:**
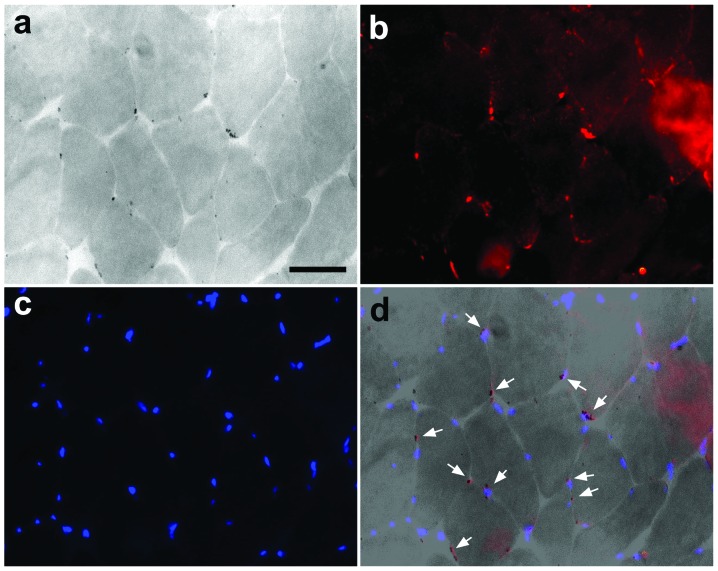
Detection of apoptotic cells in human gracilis skeletal muscle of individuals 50 years of age. Terminal deoxynucleotidyl transferase-mediated dUTP nick end-labeling (TUNEL) staining is shown in black, apoptosis-inducing factor (AIF) is colored red and DAPI is shown as blue nuclei in human gracilis skeletal muscle. (a) TUNEL-positive nuclei, (b) AIF-positive nuclei, (c) DAPI-positive nuclei, (d) overlay of TUNEL, AIF and DAPI. Arrows indicate AIF-, TUNEL- and DAPI-positive nuclei. Images were obtained at an objective magnification of ×200. Scale bars, 150 μm.

The expression of AIF and TUNEL indicated that the muscle nuclei were undergoing apoptotic changes (Fig. 5).

## Discussion

Although increased apoptosis in skeletal muscle occurs under several pathophysiological conditions (22,35,36), whether apoptosis occurs during normal aging is not clear. In this study, we examined skeletal muscle samples from individuals between 10 and 50 years of age, as well as from middle-aged individuals, when degeneration begins and self-renewal activity occurs for the maintenance of skeletal muscle cells. We demonstrated that apoptotic DNA fragmentation increased progressively with age in the human gracilis muscle. These results are in accordance with those presented in the study by Strasser *et al* (37), who found an increased incidence of apoptosis using a TUNEL assay in human rhabdosphincter skeletal muscle with age. In addition, we have previously reported the age-dependent induction of AIF and a significant increase in DNA fragmentation in the human semitendinosus skeletal muscle (33).

Caspase-3 is a central mediator of cell death as a number of apoptotic signaling pathways converge at this point (9,23). Studies have shown that caspase-3 mRNA and protein levels are elevated in the muscles of individuals of 50 years of age, although the protein level of cleaved caspase-3 was not separated in these muscles. This could be explained by the fact that in skeletal muscle, cytochrome *c* initiates the caspase-dependent apoptotic pathway, whereas AIF resides in the mitochondrion and upon stimulation, translocates to the nucleus to induce DNA fragmentation in a caspase-independent manner (29,30,38,39). AIF is a principal mediator of cell death as apoptotic signaling pathways converge at this point. In this study, we observed an increase in the mRNA level of AIF measured by RT-PCR. The muscle of individuals of 50 years of age had a higher AIF mRNA expression compared with the controls, with increased total and cleaved AIF levels in the gracilis muscle. Consistent with the mRNA data, the AIF protein content was higher in the muscles of individuals of 50 years of age than in those who were 10 years of age. Furthermore, we found that the level of AIF immunoreactivity increased with age, and a positive correlation was observed between AIF and DNA fragmentation. The relevance of caspase-independent apoptosis to age-related muscle changes was supported by the positive staining for AIF in the nucleus and the extent of apoptotic DNA fragmentation.

Bcl-2 and Bax are important apoptotic regulatory proteins that respectively inhibit and promote mitochondrial apoptogenic protein release (40,41). In addition, AIF is released from the mitochondria in response to increased levels of Bax or decreased levels of Bcl-2 (42). We observed an age-related increase in Bax and a decrease in Bcl-2 levels in skeletal muscle. Similarly, as shown in a previous study, a significant increase in Bax levels occurred in the gastrocnemius and soleus muscles of old sedentary rats, concomitant with the reduced expression of Bcl-2 in the rat soleus muscle (43). As previously demonstrated, the releae of AIF is mediated by the direct proteolysis of the protein by calpain-1 (32). In addition, the AIF and EndoG pro-apoptotic factors, which are released from the mitochondria by calpain, are expressed in muscle in the elderly. Therefore, one of the most remarkable changes that we observed in the skeletal muscle of middle-aged individuals was the increased calpain-1 levels. Depending on the relative levels of anti-apoptotic and apoptotic factors released from the mitochondria (44), the caspase-independent pathway involving AIF is activated, triggering apoptosis directly (10).

In conclusion, our study demonstrates that increased apoptosis occurs in human gracilis skeletal muscle in individuals of 50 years of age compared to those who are 10 years of age, confirming the age-related increase in apoptosis in skeletal muscle. The involvement of apoptotic pathways in the aging process was suggested by the selective changes in the expression levels of the apoptosis regulatory proteins, Bax, Bcl-2, AIF and calpain-1. This indicates the correlation between the expression of AIF and apoptosis in individuals between 10 and 50 years of age human gracilis skeletal muscle.
